# Regulation of the reserve carbohydrate metabolism by alkaline pH and calcium in *Neurospora crassa* reveals a possible cross-regulation of both signaling pathways

**DOI:** 10.1186/s12864-017-3832-1

**Published:** 2017-06-09

**Authors:** Stela Virgilio, Fernanda Barbosa Cupertino, Daniela Luz Ambrosio, Maria Célia Bertolini

**Affiliations:** Universidade Estadual Paulista (UNESP), Instituto de Química, Departamento de Bioquímica e Tecnologia Química, Araraquara, SP 14800-060 Brazil

**Keywords:** PAC-3 transcription factor, Glycogen, Trehalose, pH signaling pathway, Calcium stress, *Neurospora crassa*

## Abstract

**Background:**

Glycogen and trehalose are storage carbohydrates and their levels in microorganisms vary according to environmental conditions. In *Neurospora crassa,* alkaline pH stress highly influences glycogen levels, and in *Saccharomyces cerevisiae,* the response to pH stress also involves the calcineurin signaling pathway mediated by the Crz1 transcription factor. Recently, in yeast, pH stress response genes were identified as targets of Crz1 including genes involved in glycogen and trehalose metabolism. In this work, we present evidence that in *N. crassa* the glycogen and trehalose metabolism is modulated by alkaline pH and calcium stresses*.*

**Results:**

We demonstrated that the pH signaling pathway in *N. crassa* controls the accumulation of the reserve carbohydrates glycogen and trehalose via the PAC-3 transcription factor, which is the central regulator of the signaling pathway. The protein binds to the promoters of most of the genes encoding enzymes of glycogen and trehalose metabolism and regulates their expression. We also demonstrated that the reserve carbohydrate levels and gene expression are both modulated under calcium stress and that the response to calcium stress may involve the concerted action of PAC-3. Calcium activates growth of the Δ*pac-3* strain and influences its glycogen and trehalose accumulation. In addition, calcium stress differently regulates glycogen and trehalose metabolism in the mutant strain compared to the wild-type strain. While glycogen levels are decreased in both strains, the trehalose levels are significantly increased in the wild-type strain and not affected by calcium in the mutant strain when compared to mycelium not exposed to calcium.

**Conclusions:**

We previously reported the role of PAC-3 as a transcription factor involved in glycogen metabolism regulation by controlling the expression of the *gsn* gene, which encodes an enzyme of glycogen synthesis. In this work, we extended the investigation by studying in greater detail the effects of pH on the metabolism of the reserve carbohydrate glycogen and trehalose. We also demonstrated that calcium stress affects the reserve carbohydrate levels and the response to calcium stress may require PAC-3. Considering that the reserve carbohydrate metabolism may be subjected to different signaling pathways control, our data contribute to the understanding of the *N. crassa* responses under pH and calcium stresses.

**Electronic supplementary material:**

The online version of this article (doi:10.1186/s12864-017-3832-1) contains supplementary material, which is available to authorized users.

## Background

Glycogen and trehalose are storage carbohydrates found in many microorganisms and their contents vary dynamically, not only in response to changes in environmental conditions, but also throughout their life cycle. Glycogen is a polymer of glucose linked by α-1,4-linear and α-1,6-branched glycosidic bonds, while trehalose is a disaccharide consisting of two units of glucose linked by α-1,1-glycosidic bonds. The filamentous fungus *Neurospora crassa* accumulates glycogen during exponential growth and degrades it when its growth rate decreases [1]. Trehalose is highly accumulated in sexual spores, accounting for up to 14% of their dry weight [2]. While glycogen functions as a carbon source and energy reserve, trehalose appears to be mainly involved in stress protection in yeast and filamentous fungi [3, 4].

In eukaryotic cells, glycogen is synthesized by the action of the enzymes glycogenin, glycogen synthase, and branching enzyme, while its degradation requires glycogen phosphorylase and debranching enzyme [5]. Glycogen synthase and glycogen phosphorylase are regulated by reversible covalent modification, such that phosphorylation activates glycogen phosphorylase and inhibits glycogen synthase [6, 7]. These two enzymes are also regulated by allosterism, such that glucose-6-phosphate and adenosine monophosphate modulate glycogen synthase and glycogen phosphorylase, respectively.

Trehalose, on the other hand, is synthesized by a large complex consisting of trehalose-phosphate synthase and trehalose-phosphate phosphatase subunits [8] and is hydrolyzed by two unrelated trehalases, which differ in their optimal pH, localization and regulation. The acid trehalase (also referred to as nonregulatory trehalase) is a vacuolar enzyme, while the neutral trehalase (also referred to as regulatory trehalase) is cytosolic and is specifically phosphorylated by the cAMP-dependent protein kinase PKA [4, 9]. In *N. crassa,* both glycogen and trehalose contents vary under heat shock; glycogen is degraded under heat stress, while trehalose accumulates under such a condition [1, 10].

We have been studying the molecular mechanisms involved in glycogen and trehalose metabolism regulation in *N. crassa* under different conditions [11–14]. Using a collection of *N. crassa* mutant strains in transcription factors, we identified PAC-3 as a transcription factor likely involved in glycogen metabolism regulation [15]. Further investigation showed that PAC-3 down regulates the expression of the *gsn* gene under alkaline pH, which is the gene encoding glycogen synthase, the regulatory enzyme in glycogen synthesis. This is consistent with the low glycogen accumulation under the same condition [12]. The *Saccharomyces cerevisiae GSY1* orthologous gene is also repressed under alkaline stress [16]. PAC-3 is the homolog of the *Aspergillus nidulans* PacC and *S. cerevisiae* Rim101p proteins, which are the central regulators of the pH signaling pathway mediated by a gene cascade responsive to alkaline pH [17, 18]. In yeast, response to high pH also involves the signaling pathway mediated by the protein phosphatase calcineurin, which is triggered by an increase in cytosolic calcium. Calcineurin dephosphorylates the Crz1 transcription factor, which migrates to the nucleus leading to the regulation of calcium-responsive genes. In *S. cerevisiae,* alkali-regulated genes were also described to be dependent on calcineurin showing that the signaling pathway triggered by calcium may be involved in the pH response [16, 19]. Recently, many pH stress responsive genes were identified as targets of Crz1 in yeast, and among them genes related to glucose utilization, which include those involved in glycogen and trehalose metabolism [20]. Whether the response to both signaling pathways involves expression regulation of these genes deserves investigation. In *N. crassa,* calcineurin is an essential protein involved in hyphal morphology and required for normal growth and asexual and sexual development [21, 22]. However, its role in metabolism regulation in filamentous fungi has not yet been described.

In this work, we investigated the regulation of glycogen and trehalose metabolism under alkaline pH and calcium stresses in *N. crassa*, and we demonstrated that the accumulation of both reserve carbohydrates is modulated under both conditions. The expression of most genes encoding enzymes involved in both carbohydrate synthesis and degradation is regulated by alkaline pH and most gene promoters are bound by the PAC-3 transcription factor. We also showed that glycogen and trehalose accumulation is PAC-3 dependent and is differently regulated under low (10 mM) and high (300 mM) calcium concentration. Additionally, Ca^2+^ induces the expression of *pac-3* and regulates some genes involved in glycogen and trehalose metabolism in a wild-type strain, whose expression is regulated by PAC-3. We provide evidence that indicates the existence of a cross regulation between the pH and calcium signaling pathways. The interplay between both signaling pathways suggests an integrated regulation of the reserve carbohydrate metabolism by both pathways in *N. crassa,* which may be mediated by the PAC-3 transcription factor.

## Methods

### *Neurospora crassa* strains and growth conditions

*Neurospora crassa* FGSC#9718 (*mus-51*::*bar*), used as wild-type strain, and the FGSC#21931 (Δ*pal-1*, NCU05876), FGSC#15867 (Δ*pal-2*, NCU00317), FGSC#16419 (Δ*pal-3*, NCU03316), FGSC#22412 (Δ*pal-6*, NCU03021), FGSC#16099 (Δ*pal-8*, NCU00007), and FGSC#13378 (Δ*pal-9*, NCU01996) mutant strains were purchased from the Fungal Genetics Stock Center (FGSC, University of Missouri, Kansas City, MO, USA, http://www.fgsc.net) [23]. The constructions of the Δ*pac-3* (NCU00090) and the Δ*pac-3 pac-3* ^*+*^ (Δ*pac-3 his-3::ccg-1-mCh-pac-3*) complemented strains were described in [12, 24], respectively. The characteristics of all mutant strains and the gene and protein data were described in [24]. All strains were maintained on solid Vogel’s minimal (VM) medium, pH 5.8 [25] containing 2% sucrose at 30 °C. Conidia from 10-day old culture of wild-type, mutant, and complemented strains were suspended in sterile water and counted. For radial growth analyses, 10^7^ conidia were inoculated onto Petri dishes containing solid VM medium plus 2% sucrose either at different pH or different concentration of CaCl_2_ with or without cyclosporin A (Sigma) at 30 °C. Images of colony morphology were captured after 24 h of growth.

For pH and calcium stresses, 10^9^ conidia were first germinated in 1 L of VM medium containing 2% sucrose, pH 5.8 (time zero), at 30 °C, 200 rpm, for 24 h. After this period, the culture was filtered and the mycelia were divided in samples. One was frozen in liquid nitrogen and stored at −80 °C for further processing (control sample, not subjected to stress), while the remaining samples were individually transferred into 500 mL of fresh VM medium containing 0.5% sucrose at pH 7.8 (for alkaline pH stress) and 10 and 300 mM of CaCl_2_ (for calcium stress). Samples were harvested after 15, 30, 60, and 120 min incubation and stored at −80 °C. The mycelia samples were used for glycogen and trehalose quantification and RNA extraction.

### Glycogen, trehalose and protein quantification

Mycelia pads were extracted in lysis buffer (50 mM Tris-HCl, pH 8.0, 50 mM NaF, 1 mM EDTA, 0.5 mM PMSF, 0.1 mM TCLK, 25 mM benzamidine, and 1 μg/ml of each pepstatin and aprotinin). Cellular extracts were clarified and the supernatants were used for glycogen, trehalose and protein quantification. Glycogen content was quantified according to [26] and trehalose was quantified following the protocol described by [27], with modifications. Glycogen was precipitated with cold ethanol and digested with α-amylase and amyloglucosidase and trehalose was digested with a partially purified trehalase from *Humicola grisea* [28]. Free glucose was quantified with a glucose oxidase kit (Labtest) and glycogen and trehalose concentrations were normalized to the total protein concentration. Total protein was quantified by the [29] method using BSA as standard.

### RNA isolation and gene expression analysis

Gene expression was analyzed by RT-qPCR. Total RNA was prepared using mycelia samples according to [30] method. RNA (10 μg) from each sample was fractionated on agarose gel to assess the rRNAs integrity. RNA samples were first treated with RQ1 RNAse-free DNAse (Promega) and subjected to cDNA synthesis using the SuperScript III First Strand Synthesis kit (Invitrogen) and oligo (dT) primer according to the manufacturer’s instructions. The cDNA libraries were subjected to RT-qPCR on a StepOnePlus ™Real-Time PCR System (Applied Biosystems) using the Power SYBR® Green PCR Master Mix (Applied Biosystems) and specific primers. For genes involved in glycogen metabolism, the following primers were utilized for the *pac-3* (qPac3-F/qPac-3-R), *gsn* (qGSN-F/qGSN-R), *gbn* (qRAMIF-F/qRAMIF-R), *gnn* (qGNN-F/qGNN-R), *gpn* (qGPN-F/qGPN-R), and *gdn* (qDESRAM-F*/*qDESRAM-R) amplicons (Additional file 1: Table S1). For genes involved in trehalose metabolism, the following primers were utilized for the *tps-1* (qtps1-F/qtps1-R), *tps-1α* (qtps1alfa-F/qtps1alfa-R), *tps-2* (qtps2-F/qtps2-R), *tre-1* (qtre1-F/qtre1-R), and *tre-2* (qtre2-F*/* qtre2-R) amplicons (Additional file 1: Table S1). For *crz-1* gene expression we used the qcrz1-F/qcrz1-R primers (Additional file 1: Table S1).

Reactions were performed under the following conditions: 95 °C for 10 min, 40 cycles of 95 °C for 15 s, 60 °C for 1 min to calculate cycle threshold (Ct) values, followed by 95 °C for 15 s, 60 °C for 1 min and then 95 °C for 15 s to obtain melt curves. Data analysis was performed by the StepOne Software (Applied Biosystems) using the Comparative CT (ΔΔCT) method [31]. At least four biological replicates, with three experimental replicates per sample were performed, and reactions with non-template were used as a negative control. The fluorescent dye ROX™ was used as the passive reference to normalize the SYBR green reporter dye fluorescent signal. The PCR products were subjected to melting curves analysis to verify the presence of single amplicons. All reaction efficiencies varied from 92 to 100%. The beta-tubulin gene (β-*tub* gene, NCU04054) was used as the reference gene in pH stress analyses and the actin gene (*act* gene, NCU04173) was used as the reference gene in calcium stress.

### Chromatin immunoprecipitation-PCR assays

Chromatin immunoprecipitation assays were performed as described in [24], using mycelia from the Δ*pac-3 pac-3* ^*+*^ (Δ*pac-3 his-3::ccg-1-mCh-pac-3*) complemented strain, anti-mCherry polyclonal antibody (BioVision) and Dynabeads Protein A (Invitrogen) for immunoprecipitation. After sonication and immunoprecipitation, the DNA was quantified and 25 ng of Input DNA (IN), no Ab (N, reaction without antibody) and IP (immunoprecipitated DNA) samples were amplified by PCR using primers specific for each promoter (Additional file 1: Table S1, ChIP-PCR). Input DNA was used as a positive control of the experiment and no Ab as negative control.

PCR was performed using Phusion High-Fidelity PCR kit (Finzymes) and specific oligonucleotides for *gsn* (PacC-F/SREBP-RP2), *gpn* (pGPNNit-F2/pGPNNit-R2), *gnn* (gnnPAC3-Fp/gnnPAC3-Rp), *gbn* (branch-FP5/branch-RP5), *gdn* (DEBp-F2/DEBp-R2), *tps-1* (tresynt-Fp/tresynt-Rp), *tps-1α* (alfatre-Fp/alfatre-Rp), *tps-2* (trephosp-Fp/ trephosp-Rp), *tre-1* (tre1-Fp/tre1-Rp) and *tre-2* (tre2-Fp/tre2-Rp) promoters. A fragment of the ubiquitin gene (NCU05995), which does not have the PAC-3 motif, was amplified with the primers qUbi-F/qUbi-R and used as negative control of binding. Reactions were performed under the following conditions: 98 °C for 10 s, 25 cycles of 98 °C for 1 s, 60 °C for 5 s and 72 °C for 30 s, and then 72 °C for 5 min. The reaction products were analyzed on a 2% agarose gel and visualized by ethidium bromide. Densitometry was performed using ImageJ software [32], and the IP signals were compared to the negative control (no Ab).

## Results

### The pH-signaling pathway controls accumulation of the reserve carbohydrate glycogen and trehalose in *Neurospora crassa*

We previously identified the PAC-3 transcription factor as a putative regulator of glycogen metabolism [15], and later we showed that PAC-3 is required for proper glycogen accumulation by down-regulating the expression of the gene encoding glycogen synthase (*gsn*), the regulatory enzyme in glycogen synthesis [12]. To better investigate the role of the pH signaling pathway in the control of the reserve carbohydrate metabolism, we quantified the levels of glycogen and trehalose, another reserve carbohydrate, in mutant strains in each component of the pH pathway. The pH signaling pathway includes a pH sensor located in the plasma membrane, which, once activated by ambient alkaline pH, recruits five downstream protein components to activate the PacC and Rim101 transcription factors in *A. nidulans* and *S. cerevisiae,* respectively. *N. crassa* shares all components (PAL proteins) with the *A. nidulans* and *S. cerevisiae* pH pathways, and all of them, except PAL-9, are required for growth at alkaline pH (7.8) [24]. First, we analyzed growth of the wild-type and Δ*pac-3* strains under an acid (4.2) to alkaline (7.8) pH transition. The mutant strain showed a progressive reduction of growth under pH transition, compared to the wild-type strain, and was not able to grow at alkaline pH (7.8) (Additional file 2: Figure S1).

**Fig. 1:**
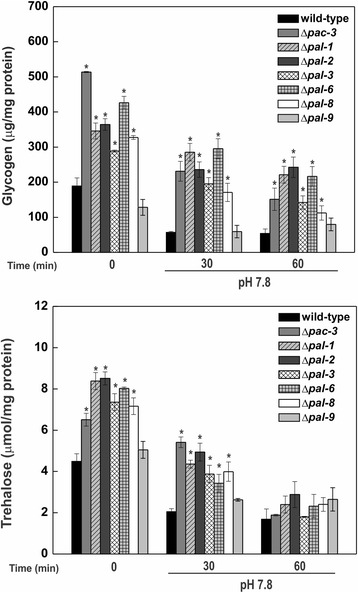
Glycogen and trehalose accumulation in the Δ*pal* mutant strains and in the wild-type strain at normal growth pH (5.8) and alkaline pH (7.8). Mycelia samples from the wild-type and Δ*pal* mutant strains cultivated at pH 5.8 and 30 °C for 24 h and shifted to pH 7.8 for 30 and 60 min were used for glycogen and trehalose quantification. Glycogen was digested with α-amylase and amyloglucosidase after precipitation with cold ethanol and trehalose was digested with a partially purified trehalase from *Humicola grisea*. Free glucose was quantified and the glycogen and trehalose concentrations were normalized to the total protein concentration. The *asterisks* indicate significant differences compared to the wild-type strain at the same condition (Student’s t-test, *P* < 0.01). All results represent the average of at least three independent experiments. *Bars* indicate the standard deviation from the biological experiments. 0, sample before pH shifting (control sample, 24 h of growth at pH 5.8)

We quantified both carbohydrates in mycelial samples from all mutant strains grown at normal growth pH (5.8) and in samples harvested at different times after shifting to pH 7.8. The levels of both carbohydrates were significantly higher in all mutant strains at pH 5.8 (time 0) compared to the wild-type strain, with the exception of the Δ*pal-9* mutant (Fig. 1). These results suggest that a functional pH signaling pathway is required for proper carbohydrate accumulation at pH 5.8. On the other hand, the transfer to pH 7.8 led to a decrease in the levels of both carbohydrates in the wild-type and mutant strains, and the repression was stronger regarding trehalose levels indicating that alkaline pH stress has a negative effect on the accumulation of both reserve carbohydrates in *N. crassa,* different from heat stress, which induces trehalose and represses glycogen accumulation [10, 14]. From these results, we observed two independent results: the requirement of an active pH signaling pathway to maintain proper levels of the reserve carbohydrates in a pH independent manner, and the repressor effect of the pathway components on the carbohydrate levels. To confirm the results, we quantified the glycogen levels in the Δ*pac-3 pac-3* ^*+*^ complemented strain at pH 5.8 and after shifting the mycelia to pH 7.8 for 1 h. The wild-type and the complemented strains showed similar glycogen levels (Additional file 3: Figure S2) indicating that *pac-3* complementation rescues the wild-type phenotype.

### Alkaline pH and PAC-3 modulate the expression of genes encoding enzymes of glycogen metabolism

**Fig. 2:**
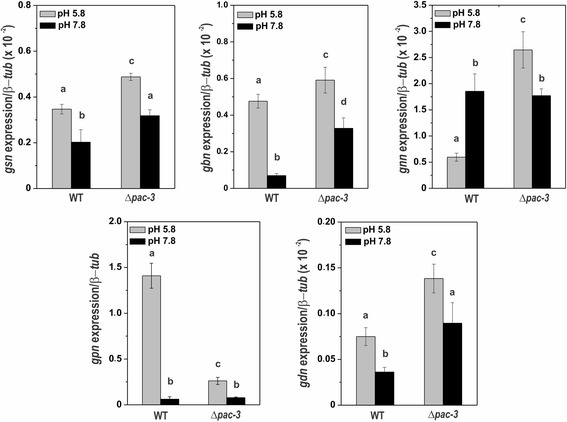
The expression of glycogenic genes in the wild-type and Δ*pac-3* mutant strains at normal growth pH (5.8) and alkaline pH (7.8). Cells from the wild-type and Δ*pac-3* strains were cultured at pH 5.8 for 24 h and shifted to pH 7.8 for 1 h. Mycelial samples were collected and used to extract total RNA. Gene expression analysis was performed by RT-qPCR in the StepOnePlus™ Real-Time PCR system (Applied Biosystems) using the Power SYBR® Green and specific primers. The β-*tub* gene was used as the reference gene, and the wild-type at pH 5.8 was used as the reference sample. At least three biological replicates were performed in triplicate, and the data were analyzed using the relative quantification standard curve method. *Bars* indicate the standard deviation from the biological experiments. a, b, c, d: *Letters above the bars* indicate statistical significance; *different letters* indicate significant difference between two samples and *similar letters* indicate no significant difference between two samples at the same or different pH (Student’s t-test, *P* < 0.01)

Since PAC-3 regulates glycogen accumulation and the expression of the *gsn* gene [12], here we investigated whether glycogen accumulation is modulated by the protein components of the pH signaling pathway. In addition, we broadly investigated the expression of all genes directly involved in glycogen metabolism. Expression of *gsn* (NCU06687, encodes glycogen synthase), *gbn* (NCU05429, encodes glycogen branching enzyme), *gnn* (NCU06698, encodes glycogenin), *gpn* (NCU07027, encodes glycogen phosphorylase), and *gdn* (NCU00743, encodes glycogen debranching enzyme) genes was analyzed in mycelia from Δ*pac-3* and wild-type strains grown at pH 5.8 for 24 h and in mycelia shifted to pH 7.8 for 1 h. All genes were repressed at pH 7.8, with the exception of *gnn* in the wild-type strain*,* which was up-regulated, consistent with the repression in glycogen accumulation under the same condition (Fig. 2). In addition, expression was dependent on the PAC-3 transcription factor under normal and alkaline pH since most of the genes were up-regulated in the Δ*pac-3* strain at both pH, and also consistent with the higher glycogen accumulation observed in the Δ*pac-3* strain at both pH (compare with Fig. 1). These results suggest that PAC-3 acts as a repressor of glycogen accumulation by down-regulating the expression of most genes encoding the enzymes required for glycogen metabolism.

**Fig. 3:**
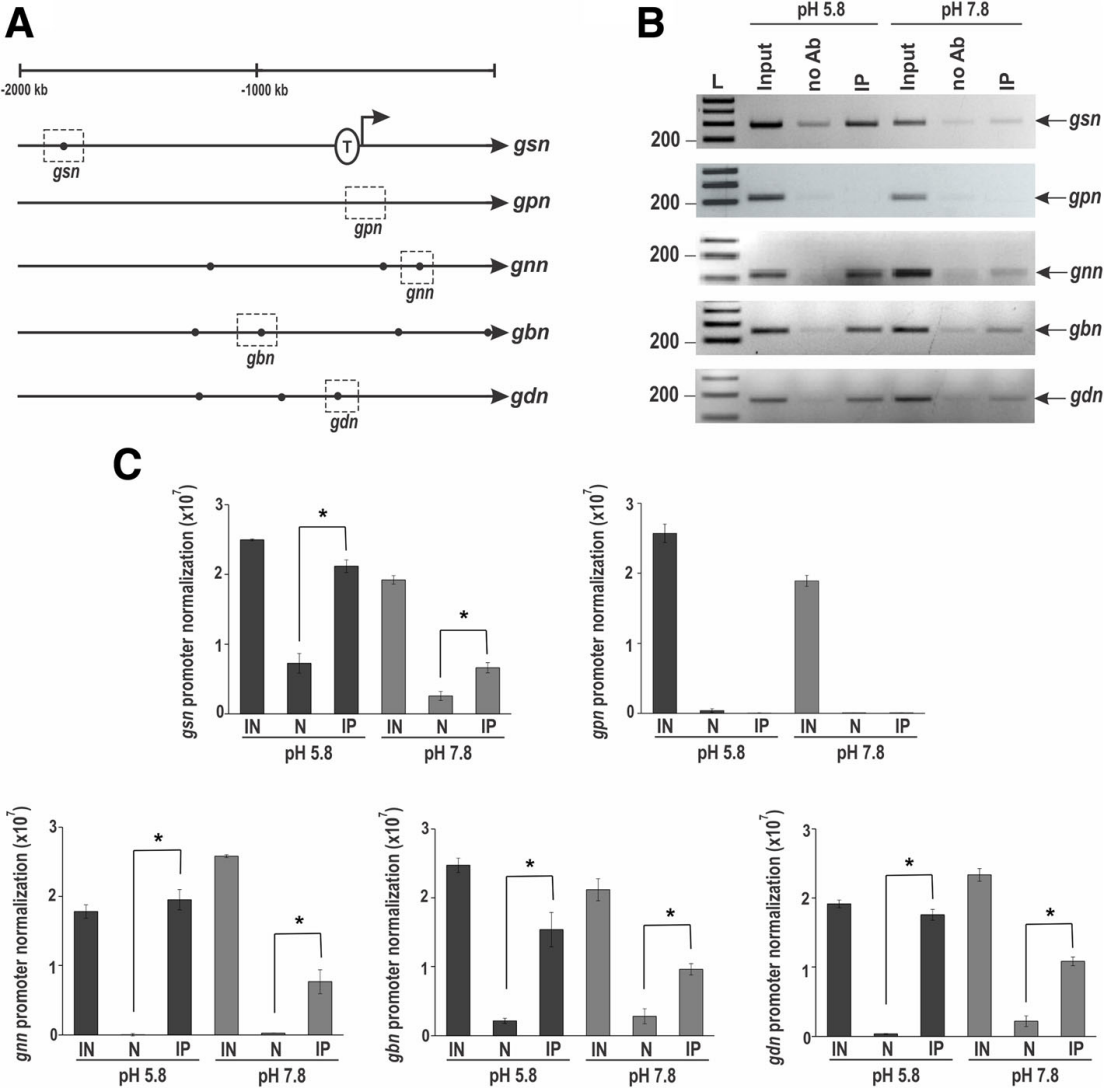
Binding of PAC-3 to the glycogenic gene promoters at normal growth pH (5.8) and alkaline pH (7.8). **a** Schematic representation of the PAC-3 DNA binding sites in the 5′-flanking regions of the glycogenic gene promoters. The *black dots* indicate the position of the PAC-3 motifs (5′-BGCCVAGV-3′) [33] identified and the *dashed boxes* indicate regions that were analyzed by ChIP-PCR. The transcription initiation site (T) in *gsn* was experimentally determined [11]. **b** Genomic DNA samples from the Δ*pac-3 pac-3* ^*+*^ complemented strain subjected to pH 7.8 stress or not subjected to it (pH 5.8) were immunoprecipitated with anti-mCherry antibody and subjected to PCR to amplify DNA fragments containing the PAC-3 motif. A DNA fragment from the *gpn* promoter, which does not have a PAC-3 motif, was used as a negative control of binding. The input DNA was used as a positive control and the non-immunoprecipitated reaction (no Ab) as the negative control. L, 1 kb DNA ladder. **c** The DNA bands from ChIP-PCR were quantified by ImageJ, and the results are shown. *Asterisks* indicate significant difference between no Ab (N) and immunoprecipitated (IP) at the same pH (Student’s t-test, *P* < 0.01). IN, Input DNA. All results represent the average of at least two independent experiments. *Bars* indicate the standard deviation from the biological experiments

An *in silico* analysis of the promoter region of the genes involved in glycogen synthesis/degradation revealed the existence of the *N. crassa* PAC-3 DNA binding site (5′-BGCCVAGV-3′) [33] in all glycogenic promoters, except in the *gpn* promoter, suggesting that PAC-3 could directly regulate the expression of these genes. A schematic representation of the gene promoters and the putative PAC-3 binding sites are shown (Fig. 3a). A ChIP-PCR assay was performed to analyze PAC-3 binding to DNA fragments containing some of these sites in vivo (shown in dashed boxes in Fig. 3a). In these experiments, we used a Δ*pac-3 pac-3* ^*+*^ complemented strain and the anti-mCherry antibody. Chromatin was collected from mycelia subjected and not to alkaline pH stress, and binding of PAC-3 to all promoters was analyzed by PCR using the oligonucleotides described in Additional file 1: Table S1 (ChIP-PCR). As a positive control of the experiments, the input DNA (IN) was analyzed, and as negative controls, we used a region of the *gpn* promoter, which lacks the PAC-3 motif and the non-immunoprecipitated reactions (no Ab, N). The DNA fragments amplified in the ChIP-PCR assays (Fig. 3b) were quantified and the results were plotted (Fig. 3c). PAC-3 was able to significantly bind to all gene promoters containing the PAC-3 motif at pH 5.8 and 7.8 (Fig. 3b and c, *P* < 0.01), and binding seemed to be more intense at pH 5.8 (Fig. 3c). The *gpn* promoter was not bound by PAC-3, as expected, confirming that this gene is not regulated by PAC-3 at pH 7.8 (see Fig. 2). Although the expression of the *gnn* gene was not influenced by PAC-3 at pH 7.8 (Fig. 2), the transcription factor bound to its gene promoter under both pH conditions (Fig. 3c). In this case, PAC-3 could bind to the promoter but not influence its expression.

Binding of PAC-3 to gene promoters and gene expression regulation by PAC-3 at pH 5.8 suggests the existence of an active pH signaling pathway at this pH in *N. crassa* and agrees with previous results showing the presence of processed, and therefore active, PAC-3 transcription factor at the same pH [24].

### Alkaline pH and PAC-3 modulate the expression of genes encoding enzymes of trehalose metabolism

**Fig. 4:**
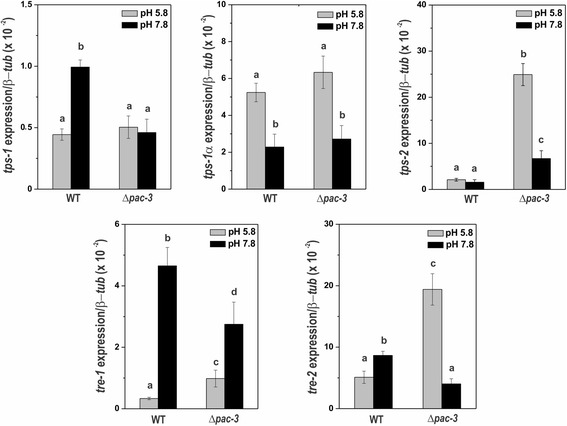
The expression of trehalose genes in the wild-type and Δ*pac-3* strains at normal growth pH (5.8) and alkaline pH (7.8). Cells from the wild-type and Δ*pac-3* strains were cultured at pH 5.8 for 24 h and shifted to pH 7.8 for 1 h. Mycelial samples were used to extract total RNA. Gene expression analysis was performed by RT-qPCR in the StepOnePlus™ Real-Time PCR system (Applied Biosystems) using the Power SYBR® Green and specific primers described in Additional file 1: Table S1. The β-*tub* gene was used as the reference gene, and the wild-type pH 5.8 was used as the reference sample. At least four biological replicates were performed in triplicate, and the data were analyzed using the relative quantification standard curve method. *Bars* indicate the standard deviation from the biological experiments. a, b, c, d: *Letters above the bars* indicate statistical significance; *different letters* indicate significant differences between two samples and *similar letters* indicate no significant difference between two samples at the same or different pH (Student’s t-test, *P* < 0.01)

As the trehalose accumulation was also influenced by PAC-3 and the PAL components of the signaling pathway under normal and alkaline pH, we analyzed the expression of the genes encoding enzymes of trehalose metabolism under the same conditions. We assayed the expression of the *tps-1* (NCU00793, encodes trehalose phosphate synthase), *tps-1α* (NCU09715, encodes alpha-trehalose phosphate synthase), *tps-2* (NCU05041, encodes trehalose phosphatase), *tre-1* (NCU00943, encodes trehalase-1) and *tre-2* (NCU04221, encodes neutral trehalase-2) genes in mycelia samples from the wild-type and Δ*pac-3* strains grown at pH 5.8 for 24 h (control) and shifted to pH 7.8 for 1 h. We observed that only *tre-1* and *tre-2* genes, which encode enzymes involved in trehalose degradation, were regulated by both pH 7.8 and PAC-3 in both strains (Fig. 4). It is interesting to observe the high expression levels of *tps-2* and *tre-2* in the Δ*pac-3* strain at pH 5.8 (Fig. 4). These results, similar to those observed for genes encoding enzymes of glycogen metabolism, show that PAC-3 and pH independently influence the glycogen and trehalose accumulation by regulating gene expression.

**Fig. 5:**
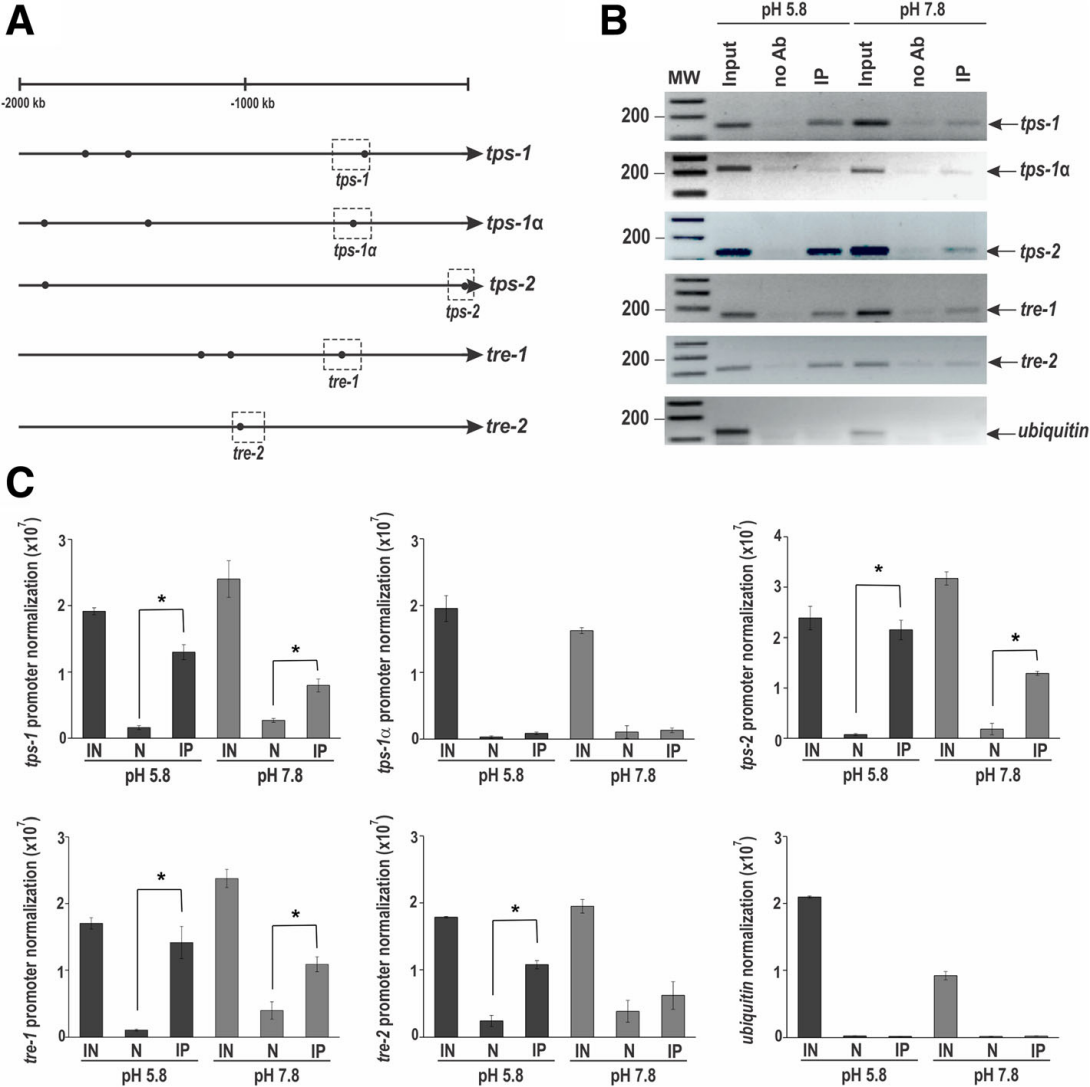
Binding of PAC-3 to the trehalose gene promoters at normal growth pH (5.8) and alkaline pH (7.8). **a** Representation of the PAC-3 motif in the trehalose gene promoters. The *black dots* indicate the position of the PAC-3 binding sites and the *dashed boxes* indicate regions that were analyzed by ChIP-PCR. **b** Genomic DNA samples from the Δ*pac-3 pac-3* ^*+*^ complemented strain subjected to pH 7.8 stress or not subjected to it (pH 5.8) were immunoprecipitated with anti-mCherry antibody and subjected to PCR to amplify DNA fragments containing the PAC-3 motif. A DNA fragment from the ubiquitin gene, which does not have a PAC-3 motif, was used as a negative control for binding. The input DNA was used as a positive control and the non-immunoprecipitated reaction (no Ab) as the negative control. L, 1 kb DNA ladder. **c** The DNA bands after ChIP-PCR were quantified by ImageJ and the results are shown. *Asterisks* indicate significant difference between no Ab (N) and immunoprecipitated (IP) at the same pH (Student’s t-test, *P* < 0.01). IN, Input DNA. All results represent the average of at least two independent experiments. *Bars* indicate the standard deviation from the biological experiments

The existence of the PAC-3 motif in all trehalose gene promoters was also confirmed by *in silico* analysis (Fig. 5a), and binding in vivo was investigated by ChIP-PCR using the same cell extracts prepared to assay the glycogen gene promoters. As a positive control, the input DNA (IN) was analyzed, and as negative controls, a fragment of the ubiquitin gene lacking the PAC-3 motif and the non-immunoprecipitated reactions (no Ab, N) were used. The DNA fragments amplified in the ChIP-PCR assays (Fig. 5b) were quantified by ImageJ and the results were plotted (Fig. 5c). PAC-3 was able to bind significantly to *tps-1*, *tps-2* and *tre-1* gene promoters at pH 5.8 and 7.8; however, the protein bound to *tre-2* promoter only at pH 5.8 (Fig. 5b and c). Although the expression of the *tps-1* gene was not influenced by PAC-3 at pH 5.8 (see Fig. 4), the transcription factor bound to its gene promoter at both pH (Fig. 5c). In this case, PAC-3 was able to bind to the promoter at pH 5.8, although the protein did not influence its expression. On the other hand, while the expression of *tre-2* was influenced by PAC-3 at both pH, PAC-3 was able to bind in vivo to its promoter only at pH 5.8 (Fig. 5). Interestingly, PAC-3 was unable to bind to *tps-1α* at both pH in agreement with the gene expression results, which showed that the *tps-1α* expression was not regulated by the transcription factor under the same conditions (see Fig. 4).

### Calcium stress affects growth of the Δ*pac-3* strain and influences its glycogen and trehalose accumulation

**Fig. 6:**
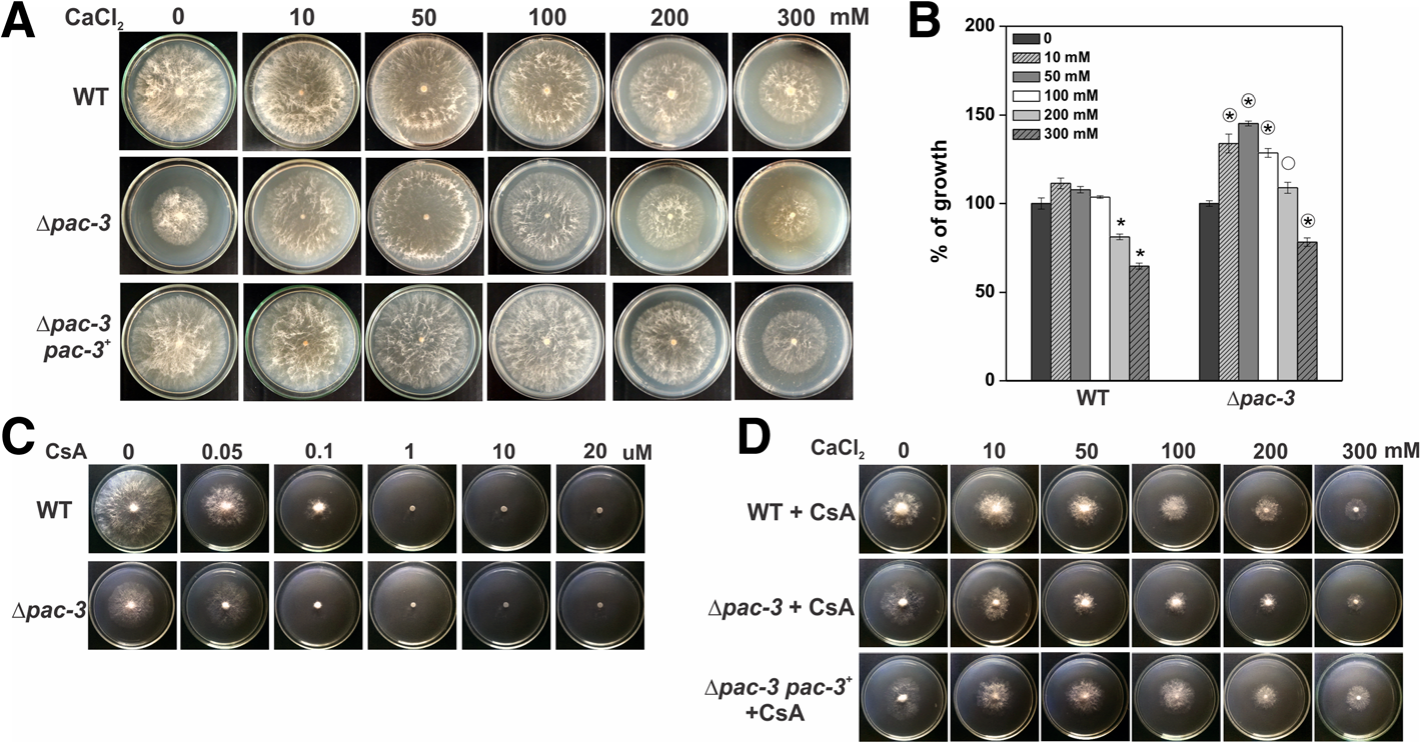
The effects of Ca^2+^ on morphological growth of the Δ*pac-3* mutant strain. **a** The wild-type, Δ*pac-3* and Δ*pac-3 pac-3* ^*+*^ strains (10^7^ conidia) were inoculated onto Petri dishes containing solid VM medium plus 2% sucrose and CaCl_2_ at final concentrations of 0, 10, 50, 100, 200 or 300 mM. Radial growth of the colonies was examined after 24 h growth at 30 °C. **b** Growth was measured and the percentage of growth was calculated taking the growth without Ca^2+^ as 100%. The results represent at least three independent experiments in duplicate. The *asterisks* indicate significant differences between the same strain cultured without Ca^2+^ and with different calcium concentrations, and the *circles* indicate significant differences compared to the wild-type strain at the same CaCl_2_ concentration (Student’s t-test, *P* < 0.01). *Error bars* indicate standard deviation. **c** The effects of 0.05, 0.1, 1, 10 and 20 μM of calcineurin inhibitor (cyclosporin A, CsA) on the wild-type and Δ*pac-3* strains radial growth. **d** The effects of different concentration of CaCl_2_ plus 0.05 μM CsA on the wild-type, Δ*pac-3* and Δ*pac-3 pac-3* ^*+*^ radial growth after 24 h at 30 °C

As previously mentioned, the signaling pathway triggered by calcium was reported to be involved in pH response in *S. cerevisiae* [16, 19, 20]*.* In this work, we investigated whether the responses to both signaling pathways are involved in the regulation of the *N. crassa* glycogen and trehalose metabolism. First, we investigated the calcium response in the wild-type, Δ*pac-3* and Δ*pac-3 pac-3* ^*+*^ strains by analyzing the growth of the strains in the presence of increasing calcium concentration, from 10 to 300 mM CaCl_2_. Increased calcium concentration differently influenced growth of the wild-type and mutant strains; the absence of a functional pH signaling significantly influenced the response to calcium stress. While growth of the wild-type strain was significantly reduced in the presence of a CaCl_2_ concentration above 200 mM, growth of the mutant strain significantly increased up to 100 mM calcium and reduced only in the presence of the highest concentration tested (300 mM). Growing the complemented strain under the same conditions restored the wild-type phenotype, confirming that the calcium effects on growth observed in the mutant strain are indeed due to the *pac-3* deletion (Fig. 6a and b).

As the intracellular calcium response involves activation of the protein phosphatase calcineurin, which is inactivated by cyclosporin A (CsA), we analyzed growth of the strains in the presence of CsA. Addition of CsA led to a strong impact in the wild-type strain growth, but not in the mutant strain, at the lowest concentration used (0.05 μM), and a complete growth inhibition of the wild-type and mutant strains at concentrations above 0.1 μM (Fig. 6c) indicating that the calcium response requires calcineurin activity. We confirmed that the calcium response was only calcineurin-dependent by analyzing the effect of a sub-inhibitory concentration of cyclosporine (0.05 μM) on the growth of wild-type, mutant and complemented strains in the presence of increased calcium concentration. The combination of cyclosporine and increasing calcium concentration did not lead to an additive effect of calcium at low concentration. This suggests that the response to calcium stress only involves the calcineurin pathway (Fig. 6d).

**Fig. 7:**
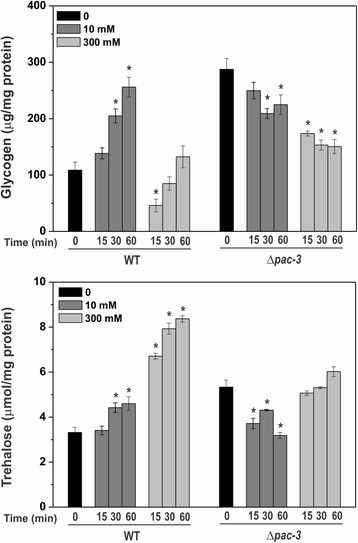
Glycogen and trehalose accumulation in the wild-type and Δ*pac-3* mutant strains at 10 and 300 mM of CaCl_2_. Mycelial samples from the wild-type and Δ*pac-3* mutant strain cultured in the absence of Ca^2+^ (zero) at 30 °C for 24 h and shifted to calcium stress (10 mM or 300 mM of CaCl_2_) for 15, 30 and 60 min were used for glycogen and trehalose quantification. The *asterisks* indicate significant differences compared to the zero sample in the wild-type or mutant strains (Student’s t-test, *P* < 0.01). All results represent the average of at least four independent experiments. *Bars* indicate the standard deviation from the biological experiments

In Fig. 1, we demonstrated the higher glycogen and trehalose levels in the Δ*pac-3* strain compared to the wild-type strain at pH 5.8 and 7.8. We previously demonstrated that the pH signaling pathway in *N. crassa* may be active at pH 5.8 [24]; therefore, the results of this work indicate that proper glycogen and trehalose levels require an active pH signaling pathway in a pH-independent manner. Since response to alkaline pH also involves the calcium signaling pathway among additional signaling pathways in *S. cerevisiae* [18], we next investigated the glycogen and trehalose levels in wild-type and Δ*pac-3* strains subjected to calcium stress at pH 5.8 using calcium concentrations based on the results shown in Fig. 6b. As demonstrated in the figure, low calcium concentration (10 mM) increases growth of the Δ*pac-3* strain while high concentration (300 mM) reduces growth of the same strain. Low calcium concentration (10 mM) results in similar responses regarding glycogen and trehalose accumulation in both strains; the levels of both carbohydrates were significantly increased in the wild-type strain and significantly reduced in the Δ*pac-3* strain (Fig. 7). However, under high calcium concentration (300 mM), we observed divergent results regarding accumulation of both carbohydrates in the strains. While glycogen levels were decreased in both strains, the trehalose levels were significantly increased in the wild-type strain and not affected by calcium in the mutant strain when compared to mycelium not exposed to calcium (Fig. 7). In addition, the trehalose levels in the mutant strain under high calcium concentration were higher than those observed at low concentration. In summary, the results suggest that both signaling pathways may contribute to maintain the proper levels of the reserve carbohydrate in *N. crassa,* and that the response to calcium stress may involve the concerted action of PAC-3.

### Involvement of calcium and pH stresses in the expression regulation of genes encoding enzymes of both reserve carbohydrates

**Fig. 8:**
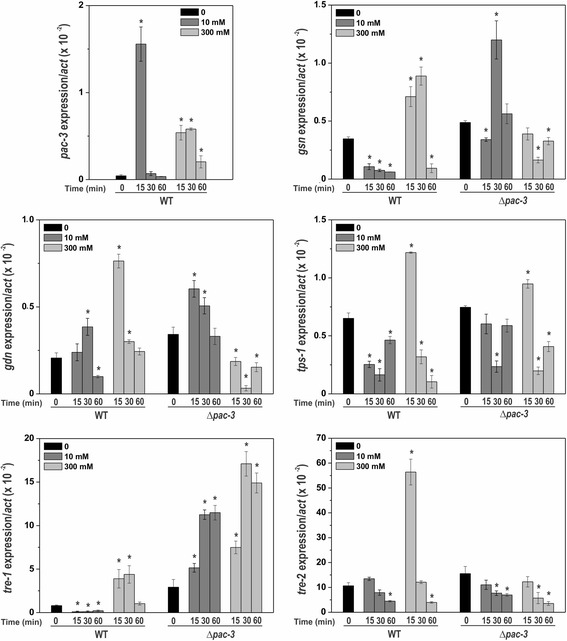
The influence of Ca^2+^ on the expression levels of some genes related to glycogen and trehalose regulation in the wild-type and Δ*pac-3* strains. Cells from the wild-type and Δ*pac-3* strains were cultured at pH 5.8 (0) for 24 h and shifted to 10 mM or 300 mM CaCl_2_ for 15, 30 and 60 min. Mycelial samples were used to extract total RNA. Gene expression analysis was performed by RT-qPCR in the StepOnePlus™ Real-Time PCR system (Applied Biosystems) using the Power SYBR® Green and specific primers (Additional file 1: Table S1, qPCR). The actin gene was used as the reference gene, and the zero sample in the wild-type was used as the reference sample. At least four biological replicates were performed, and the data were analyzed using the relative quantification standard curve method. *Bars* indicate the standard deviation from the biological experiments. The *asterisks* indicate significant differences compared to the zero sample in the wild-type or mutant strains (Student’s t-test, *P* < 0.01)

To better characterize the involvement of both signaling pathways in the reserve carbohydrate accumulation, we analyzed the expression of some genes encoding enzymes of the metabolism of both carbohydrates. First, we analyzed the *pac-3* expression in the wild-type strain under both calcium concentrations (10 and 300 mM). A rapid and intense response was observed at low calcium concentration and high transcript levels were observed at high concentration (Fig. 8) indicating that calcium influences the *pac-3* transcript levels independently of the concentration. We investigated the role of calcium and pH stresses in the expression regulation of genes encoding enzymes of the glycogen and trehalose metabolism. We used the same calcium concentrations previously used and we analyzed the expression of the some genes of glycogen metabolism (*gsn* and *gdn*) and trehalose metabolism (*tps-1*, *tre-1* and *tre-2*), which are regulated by PAC-3 at alkaline pH (Figs. 2 and 4). The results show that calcium concentration differently influences the expression of all genes (Fig. 8). In general, in the wild-type strain, low calcium concentration (10 mM) represses gene expression while high calcium concentration (300 mM) induces gene expression. On the other hand, in the mutant strain, we observed divergent results when both calcium concentrations are compared. At low concentration, expression of some genes was activated, such as *gsn, gdn,* and *tre-1* while the expression of *tre-2* was significantly reduced. In the same way, at high concentration, expression of some genes was repressed, such as *gsn, gdn*, *tps-1,* and *tre-2* while *tre-1* was significantly overexpressed. It is interesting to observe the high expression levels of the *tre-1* and *tre-2* genes, which encode the trehalase-1 and trehalase-2 enzymes, respectively, both involved in trehalose hydrolysis. It is important to emphasize that the genes assayed encode enzymes involved in the carbohydrates synthesis (*gsn* and *tps-*1) and degradation (*gdn, tre-1* and *tre-2*), and we should consider that additional ways of regulation, such as the enzymatic activity regulation could also participate in the controlling of the levels of both carbohydrates.

**Fig. 9:**
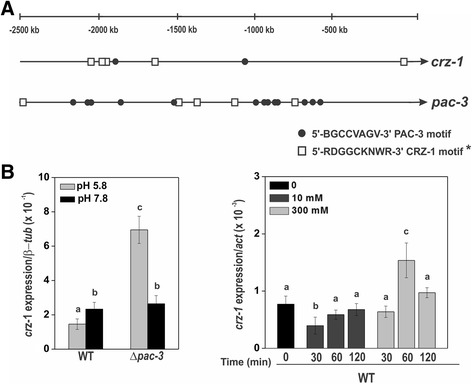
The influence of pH and Ca^2+^ on the *crz-1* expression. **a** Schematic representation of the *crz-1* and *pac-1* promoters and the respective transcription factors DNA binding sites. The *black dots* and *square boxes* indicate the position of PAC-3 and CRZ-1 motifs, respectively. **b** Expression of *crz-1* gene in Δ*pac-3* and wild-type strains under normal and alkaline pH (*left graph*) and under different CaCl_2_ concentration (*right graph*). Cells from both strains were cultured at pH 5.8 during 24 h and shifted to pH 7.8 during 1 h. Cells from the wild-type strain were cultured at normal pH (0) for 24 h and shifted to 10 or 300 mM CaCl_2_ for 30, 60 and 120 min. Mycelial samples were collected and used to extract total RNA. Gene expression analysis was performed by RT-qPCR in the StepOnePlus™ Real-Time PCR system (Applied Biosystems) using the Power SYBR® Green and specific primers (Additional file 1: Table S1, qPCR). The *tub-2* and *act* genes were used as the reference genes in the pH and calcium experiments, respectively. At least three biological replicates were run and the data were analyzed in the relative quantification standard curve method. a, b, c: *Letters above bars* indicate statistical significance; *different letters* indicate significant differences between two samples and equal letters indicate no significant difference between two samples in the same or different pH (Student’s *t*-test, *P* < 0.01). * *N. crassa* DNA binding site according to Weirauch et al. [33]

**Fig. 10:**
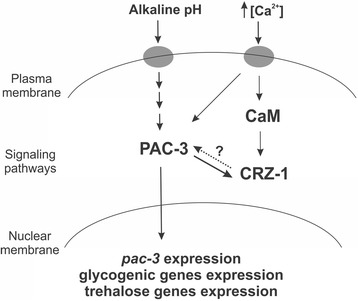
Schematic representation of the cross-regulation between alkaline pH and calcium signaling pathways in *N. crassa.* The regulatory mechanism is presented in the text. *Dotted line* and *question mark* between PAC-3 and CRZ-1 indicate an interaction likely existing, but not investigated in this work

Although the results of gene expression (Fig. 8) were not consistent with the glycogen levels observed under different calcium concentrations (Fig. 7), we may conclude that gene expression was significantly influenced by calcium concentration and PAC-3. This suggests the existence of a cross-regulation of both signaling pathways for the proper regulation of glycogen and trehalose metabolism. Since Crz1 is described as the transcription factor activated by calcineurin under calcium stress [34], we identified the NCU07952 gene product (named CRZ-1) as the putative *N. crassa* homolog protein. Sequence alignment of the *N. crassa* CRZ-1, *Aspergillus fumigatus* and *A. nidulans* CrzA and *Trichoderma reesei* CRZ-1 was performed and showed that the *N. crassa* protein shares the conserved calcineurin interaction site and the zinc finger DNA binding domain (Additional file 4: Figure S3). To investigate the cross-regulation of both signaling pathways, we first analyzed the *crz-1* and *pac-3* promoters (2500 bp upstream the ATG start codon), and a high number of DNA binding sites for both transcription factors were  identified in their promoters (Fig. 9a). *crz-1* expression was significantly increased only under high calcium concentration (300 mM CaCl_2_) indicating that CRZ-1 is a transcription factor responsive to calcium stress. In addition, *crz-1* expression was modulated by alkaline pH and by the PAC-3 transcription factor only under pH 5.8 (Fig. 9b). These data suggest an integrated regulation of both signaling pathways in *N. crassa,* which is likely required to maintain proper glycogen and trehalose levels. Based on the results, we propose a model (Fig. 10) in which high calcium concentration activates the CRZ-1 (Fig. 9), likely via calmodulin (CaM), and PAC-3 (Fig. 8) transcription factors. Alkaline pH also activates PAC-3, as previously demonstrated [24], and PAC-3 regulates *crz-1* expression under pH 5.8. We propose that the interplay between both signaling pathways results in the regulation of both transcription factors*,* and in the regulation of genes encoding enzymes involved in the reserve carbohydrate metabolism glycogen and trehalose.

## Discussion

Environmental alkalinization strongly influences a diverse set of biological processes by involving the activation of multiple signaling pathways, which results in the coordinated action of numerous proteins [18, 35]. In *S. cerevisiae* and *Candida albicans,* the responses to high pH stress, mediated by the PacC/Rim101p transcription factor, respectively, have been reported and numerous cellular processes have been described, indicating that medium alkalinization has broad effects on the cell biology [16, 19, 36, 37]. Among the numerous processes identified, the carbohydrate metabolism is strongly affected by alkalinization of the medium [19, 38]. In addition, in the yeast *S. cerevisiae,* the proper regulation of genes of the carbohydrate metabolism under medium alkalinization may also involve additional controls, including the Snf1 protein kinase [39] and the calcineurin signaling pathway [20]. We previously reported that ambient pH controls glycogen accumulation in *N. crassa* by regulating the *gsn* expression [12], and in this work, we extended the investigation by studying in more details the effects of pH and calcium stresses on the metabolism of the reserve carbohydrate glycogen and trehalose*.*

We demonstrated here that an active pH signaling pathway is required for proper regulation of glycogen and trehalose accumulation irrespective of pH (normal growth pH and alkaline pH). The Δ*pac-3* and Δ*pal* strains hyper accumulated glycogen and trehalose at normal and alkaline pH when compared to the wild-type strain indicating a repressor role of PAC-3, which is activated by this signaling pathway, on the carbohydrate metabolism. We also demonstrated that proper carbohydrate levels were influenced by the pathway components (PAL proteins) showing that an active signaling pathway is required to maintain normal carbohydrate levels. In addition, we showed here that PAC-3 regulates the expression of most genes involved in the synthesis and degradation of glycogen and trehalose, most likely due to the ability of PAC-3 to bind in vivo to most gene promoters at both pH conditions, regulating their expression. An interesting point is that the pH signaling pathway is involved in the regulation of the glycogen and trehalose levels at pH 5.8. As previously described, induction of genes involved in carbohydrate metabolism was observed in *S. cerevisiae*; however, only at pH 8.0 [19, 38]. Since we previously reported the existence of a processed PAC-3, and therefore a likely active protein at pH 5.8 [24], we hereby suggest that the regulation results from an active pH signaling pathway at this pH. Additionally, we may also suggest the existence of an active PAC-3 in the absence of *pal* signaling, being functional in a pH-independent manner, either as a full-length or proteolyzed protein. However, we cannot preclude the existence of additional proteins being involved in such regulation, similar to the PacX protein in *A. nidulans* [40]. This protein was reported to play a role in *pacC* gene repression. In addition, different signaling pathways could be involved establishing a signaling network that results in hyper accumulation of the reserve carbohydrates. Previous work has demonstrated the involvement of the cAMP-PKA signaling pathway [26] and the SNF-1 protein kinase [41] in glycogen metabolism regulation in *N. crassa*, which are described as involved in the response of *S. cerevisiae* to high pH [39, 42]. We hypothesize that both of these pathways likely contribute to the pH signaling pathway in *N. crassa* in order to maintain proper levels of the reserve carbohydrate in a pH-independent response.

### Calcium and pH signaling pathways may cooperate to maintain proper glycogen and trehalose levels in *N. crassa*

In *S. cerevisiae* and *C. albicans,* it has been demonstrated that different signaling pathways cross talk to regulate the response to alkaline stress. One is the calcineurin pathway; some genes responsive to alkaline pH are also regulated by the phosphatase calcineurin [16, 43–45]. Increased calcium concentration is an environmental condition that activates calcineurin, which dephosphorylates the Crz1 transcription factor, resulting in its activation and nuclear localization [46]. Although in yeast, both pathways are involved in the adaptation to alkaline pH, their interaction in the regulation of specific cellular processes in filamentous fungi has not yet been described. In this work, we present evidence that accumulation of the carbohydrates glycogen and trehalose and the expression of genes encoding enzymes involved in their metabolism are influenced by calcium concentration and that the response to calcium stress is also influenced by PAC-3. We also demonstrate that the calcium concentration differently impacts the carbohydrates levels in the wild-type and Δ*pac-3* mutant strains. While low calcium concentration (10 mM) increases the trehalose and glycogen levels in the wild-type strain, their levels decrease in the mutant strain. On the other hand, high calcium concentration (300 mM) significantly increases the trehalose levels in the wild-type strain but has no effect in the mutant strain. In addition, high calcium concentration significantly decreases glycogen levels in the mutant strain while having no effect in the wild-type strain. This suggests that the pH signaling and the calcineurin pathways may interact to properly regulate the reserve carbohydrate metabolism in *N. crassa*.

Previous works reported that yeast genes related to metabolism, including glycogen and trehalose metabolism, appear to be regulated by multiple regulatory pathways, such as those involving the Snf1 protein kinase, the Msn2/Msn4 transcription factors, and the calcineurin pathway [18]. We described the involvement of the SNF-1 pathway in the regulation of glycogen metabolism in *N. crassa;* however the regulatory mechanism seems to be the opposite of the one described for *S. cerevisiae* [41]*.* While *snf1* yeast mutants are not able to accumulate glycogen [47], the *N. crassa ∆snf-1* strain accumulated higher glycogen levels than the wild-type strain. The zinc-finger Msn2p/Msn4p transcription factors, which regulate the expression of stress-responsive genes by binding to the stress response element (STRE), also regulate genes induced by alkaline pH stress [42]. However, *N. crassa* apparently lacks real Msn2/4p orthologs, which suggests that the stress responses in microorganisms may have evolved differently. We recently identified the SEB-1 transcription factor as a STRE-binding protein in *N. crassa* [14]*.* This protein also regulates the metabolism of the reserve carbohydrates glycogen and trehalose under heat stress, and the ∆*seb-1* strain is sensitive to alkaline pH stress. However, the involvement of SEB-1 in the regulation of the glycogen and trehalose metabolism under alkaline stress in *N. crassa* requires further investigation. This could reveal new data regarding the modulation of this process by these signaling pathways in this filamentous fungus.

The requirement of the calcineurin pathway in the alkaline pH response of genes involved in glucose utilization in yeast, including genes encoding enzymes of glycogen and trehalose, has been previously described [48]. More recently, the contribution of the calcineurin pathway was confirmed by demonstrating the recruiting of the Crz1 transcription factor via the Calcineurin Dependent Response Element (CDRE) in the promoters of the same genes in response to high pH stress [20]. In *S. cerevisiae,* the response to alkaline stress includes the increase of cytosolic calcium, which leads to the activation of the Crz1 transcription factor via dephosphorylation by the phosphatase calcineurin [46]. In this work, we demonstrated that extracellular calcium influences glycogen and trehalose metabolism, and that PAC-3 influences in the calcium response. We also identified the *N. crassa* Crz1 orthologous protein (CRZ-1) and demonstrated that gene expression is induced by extracellular calcium and is modulated by pH and by the PAC-3 transcription factor. Since the *pac-3* promoter and most of the glycogen and trehalose genes analyzed in this work possess CDRE at their promoter regions, the role of the *N. crassa* Crz1 orthologous protein in cooperation with the pH signaling pathway to regulate glycogen and trehalose metabolism should be investigated. Although not yet described in the literature, we may not preclude the existence of additional transcription factors activated by extracellular calcium, which could also mediate such a response.

The results shown in this work demonstrate that in *N. crassa,* alkaline pH controls glycogen and trehalose accumulation and that the signaling pathway triggered by calcium stress might cooperate in the regulation. Although we have investigated these two signaling pathways here, it is an attempt to speculate whether the proper regulation of the reserve carbohydrates in *N. crassa* may involve the interaction of different signaling pathways.

## Conclusions

The effect of pH and calcium stresses on the regulation of the reserve carbohydrates glycogen and trehalose was investigated. The levels and the expression of genes encoding enzymes of both carbohydrates were affected by alkaline pH and calcium concentration. Our data confirm and extend previous results reported in *N. crassa* regarding the control of both reserve carbohydrates, and we speculate that the proper levels of both carbohydrates are under the control of different signaling pathways.

## Additional files

Additional file 1: Table S1. Oligonucleotides used in this study. (DOCX 29 kb)

Additional file 2: Figure S1. Morphological analyses of the wild-type and Δ*pac-3* mutant strains under different pH conditions. The strains (10^7^ conidia) were inoculated onto Petri dishes containing solid VM medium plus 2% sucrose from pH 4.2 (acid condition) to pH 7.8 (alkaline condition) at 30 °C. Images of colony morphology were captured after 24 h. Apical extension was measured in centimeters and is shown in the table below. The results represent at least two independent experiments in duplicate. The asterisks indicate the significant difference between wild-type and mutant strains at the same pH (Student’s t-test, *P* < 0.01). (TIFF 3061 kb)

Additional file 3: Figure S2. Glycogen quantification in the wild-type, Δ*pac-3* mutant and Δ*pac-3 pac-3* ^*+*^ complemented strains at normal growth pH (5.8) and alkaline pH (7.8). Mycelial samples cultured at pH 5.8 (zero) at 30 °C for 24 h and shifted to pH 7.8 for 1 h were used for glycogen quantification. The asterisks for the Δ*pac-3* data indicate significant differences compared to the wild-type strain at the same condition (Student’s t-test, *P* < 0.01). The results represent the average of three independent experiments. Bars indicate the standard deviation from the biological experiments. (TIFF 317 kb)

Additional file 4: Figure S3. Multiple sequence alignment of the *N. crassa* protein codified by the ORF NCU07952 and the CrzA proteins from *A. fumigatus* (XP_750439.1) and *A. nidulans* (BAE94327.1), and the CRZ-1 protein from *Trichoderma reesei* (ETS01683.1). The C_2_H_2_ zinc finger DNA binding domain at the C-terminus is highlighted by asterisks and the putative calcineurin interaction site is indicated by an upper line. ClustalW (http://www.ebi.ac.uk/Tools/msa/clustalw2/) was used for sequence alignment. Identical amino acids are shaded in black and conserved amino acids are shown in gray. (DOC 48 kb)

## Abbreviations

FGSC: Fungal genetics stock center
VM: Vogel’s minimal medium
CsA: Cyclosporin A
BSA: Bovine serum albumin
ChIP: Chromatin immunoprecipitation
CaM: Calmodulin

## References

[1] de Paula R, de Pinho CA, Terenzi HF, Bertolini MC (2002). Cloning and molecular characterization of the *gsn* cDNA encoding glycogen synthase in *Neurospora crassa*. Mol Gen Genomics.

[2] Hecker LI, Sussman AS (1973). Localization of trehalase in the ascospores of Neurospora: relation to ascospore dormancy and germination. J Bacteriol.

[3] Wiemken A (1990). Trehalose in yeast, stress protectant rather than reserve carbohydrate. Antonie Van Leeuwenhoek.

[4] Jorge JA, Polizeli ML, Thevelein JM, Terenzi HF (1997). Trehalases and trehalose hydrolysis in fungi. FEMS Microbiol Lett.

[5] Roach PJ, Skurat AV, Harris RA. Regulation of glycogen metabolism. In: Jefferson LS, Cherrington AD, editors. Handbook of physiology. Vol. 2: The endocrine pancreas and regulation of metabolism 2011, 609-47. First published in print 2001. doi:10.1002/cphy.cp070219.

[6] Téllez-Iñón MT, Terenzi H, Torres HN. Interconvertible forms of glycogen synthetase in *Neurospora crassa*. Biochim Biophys Acta. 1969;191:765–8. 10.1016/0005-2744(69)90383-0 5363997

[7] Fletterick RJ, Madsen NB (1980). The structures and related functions of phosphorylase a. Annu Rev Biochem.

[8] Bell W, Sun W, Hohmann S, Wera S, Reinders A, De Virgilio C (1998). Composition and functional analysis of the *Saccharomyces cerevisiae* trehalose synthase complex. J Biol Chem.

[9] Thevelein JM (1984). Regulation of trehalose mobilization in fungi. Microbiol Rev.

[10] Noventa-Jordão MA, Polizeli MLTM, Bonini BM, Jorge JA, Terenzi HF (1996). Effects of temperature shifts on the activities of *Neurospora crassa* glycogen synthase, glycogen phosphorylase and trehalose-6-phosphate synthase. FEBS Lett.

[11] Freitas FZ, Bertolini MC (2004). Genomic organization of the *Neurospora crassa gsn* gene. Possible involvement of the STRE and HSE elements in the modulation of gene transcription during heat shock. Mol Gen Genomics.

[12] Cupertino FB, Freitas FZ, de Paula RM, Bertolini MC. Ambient pH controls glycogen levels by regulating glycogen synthase gene expression in *Neurospora crassa*. New insights into the pH signaling pathway. PLoS One. 2012;7 doi:10.1371/journal.pone.0044258. 10.1371/journal.pone.0044258 PMC3432076 22952943

[13] Cupertino FB, Virgilio S, Freitas FZ, Candido Tde S, Bertolini MC (2015). Regulation of glycogen metabolism by the CRE-1, RCO-1 and RCM-1 proteins in *Neurospora crassa*. The role of CRE-1 as the central transcriptional regulator. Fungal Genet Biol.

[14] Freitas ZF, Virgilio S, Cupertino FB, Kowbel DJ, Fioramonte M, Gozzo FC (2016). The SEB-1 transcription factor binds to the STRE motif in *Neurospora crassa* and regulates a variety of cellular processes including the stress response and reserve carbohydrate metabolism. G3 (Bethesda).

[15] Gonçalves RD, Cupertino FB, Freitas FZ, Luchessi AD, Bertolini MC. A genome-wide screen for *Neurospora crassa* transcription factors regulating glycogen metabolism. Mol Cell Proteomics. 2011; doi:10.1074/mcp.M111.007963. 10.1074/mcp.M111.007963 PMC3226398 21768394

[16] Serrano R, Ruiz A, Bernal D, Chambers JR, Ariño J (2002). The transcriptional response to alkaline pH in *Saccharomyces cerevisiae*: evidence for calcium-mediated signalling. Mol Microbiol.

[17] Peñalva MA, Tilburn J, Bignell E, Arst HN (2008). Ambient pH gene regulation in fungi: making connections. Trends Microbiol.

[18] Serra-Cardona A, Canadell D, Ariño J (2015). Coordinate responses to alkaline pH stress in budding yeast. Microbial Cell.

[19] Viladevall L, Serrano R, Ruiz A, Domenech G, Giraldo J, Barceló A (2004). Characterization of the calcium-mediated response to alkaline stress in *Saccharomyces cerevisiae*. J Biol Chem.

[20] Roque A, Petrezsélyová S, Serra-Cardona A, Ariño J (2016). Genome-wide recruitment profiling of transcription factor Crz1 in response to high pH stress. BMC Genomics.

[21] Prokisch H, Yarden O, Dieminger M, Tropschug M, Barthelmess IB (1997). Impairment of calcineurin function in *Neurospora crassa* reveals its essential role hyphal growth, morphology and maintenance of the apical Ca^2+^ gradient. Mol Gen Genet.

[22] Tamuli R, Deka R, Borkovich KA (2016). Calcineurin subunits A and B interact to regulate growth and asexual and sexual development in *Neurospora crassa*. PLoS One.

[23] McCluskey K (2003). The fungal genetics stock center: from molds to molecules. Adv Appl Microbiol.

[24] Virgilio S, Cupertino FB, Bernardes NE, Freitas FZ, Takeda AAS, Fontes MRM, et al. Molecular components of the *Neurospora crassa* pH signaling pathway and their regulation by pH and the PAC-3 transcription factor. PLoS One. 2016; doi:10.1371/journal.pone.0161659. 10.1371/journal.pone.0161659 PMC4996508 27557053

[25] Vogel HJ (1956). A convenient growth medium for *Neurospora crassa* (medium N). Microbiol Gene Bull.

[26] Freitas FZ, de Paula RM, Barbosa LCB, Terenzi HF, Bertolini MC (2010). cAMP signaling pathway controls glycogen metabolism in *Neurospora crassa* by regulating the glycogen synthase gene expression and phosphorylation. Fungal Genet Biol.

[27] Neves MJ, Jorge JA, François JM, Terenzi HF (1991). Effects of heat shock on the level of trehalose and glycogen, and on the induction of thermotolerance in *Neurospora crassa*. FEBS Lett.

[28] Zimmermann NA, Terenzi HF, Jorge JA (1990). Purification and properties of an extracellular conidial trehalase from *Humicola grisea* var. *thermoidea*. Biochim Biophys Acta.

[29] Hartree EF (1972). Determination of protein: a modification of the Lowry method that gives a linear photometric response. Anal Biochem.

[30] Sokolovsky V, Kaldenhoff R, Ricci M, Russo VEA (1990). Fast and reliable mini-prep RNA extraction from *Neurospora crassa*. Fungal Genet Newsl.

[31] Livak KJ, Schmittgen TD (2001). Analysis of relative gene expression data using real-time quantitative PCR and the 2(−Delta Delta C(T)) method. Methods.

[32] Abramoff MD, Magalhaes PJ, Ram SJ (2004). Image processing with image. J Biophotonics Intern.

[33] Weirauch MT, Yang A, Albu M, Cote AG, Montenegro-Montero A, Drewe P (2014). Determination and inference of eukaryotic transcription factor sequence specificity. Cell.

[34] Stathopoulos-Gerontides A, Guo JJ, Cyert MS (1999). Yeast calcineurin regulates nuclear localization of the Crz1p transcription factor through dephosphorylation. Genes Dev.

[35] Ariño J (2010). Integrative responses to high pH stress in *S. cerevisiae*. OMICS.

[36] Lamb TM, Mitchell AP (2003). The transcription factor Rim101p governs ion tolerance and cell differentiation by direct repression of the regulatory genes NRG1 and SMP1 in *Saccharomyces cerevisiae*. Mol Cell Biol.

[37] Bensen ES, Martin SJ, Li M, Berman J, Davis DA (2004). Transcriptional profiling in *Candida albicans* reveals new adaptive responses to extracellular pH and functions for Rim101p. Mol Microbiol.

[38] Canadell D, García-Martínez J, Alepuz P, Pérez-Ortín JE, Ariño J (1849). Impact of high pH stress on yeast gene expression: a comprehensive analysis of mRNA turnover during stress responses. Biochim Biophys Acta.

[39] Casamayor A, Serrano R, Platara M, Casado C, Ruiz A, Ariño J (2012). The role of the Snf1 kinase in the adaptive response of *Saccharomyces cerevisiae* to alkaline pH stress. Biochem J.

[40] Bussink HJ, Bignell EM, Múnera-Huertas T, Lucena-Agell D, Scazzocchio C, Espeso EA (2015). Refining the pH response in *Aspergillus nidulans*: a modulatory triad involving PacX, a novel zinc binuclear cluster protein. Mol Microbiol.

[41] Candido TS, Gonçalves RD, Felício AP, Freitas FZ, Cupertino FB, De Carvalho AC (2014). A protein kinase screen of *Neurospora crassa* mutant strains reveals that the SNF1 protein kinase promotes glycogen synthase phosphorylation. Biochem J.

[42] Casado C, González A, Platara M, Ruiz A, Ariño J (2011). The role of the protein kinase A pathway in the response to alkaline pH stress in yeast. Biochem J.

[43] Kullas AL, Martin SJ, Davis D (2007). Adaptation to environmental pH: integrating the Rim101 and calcineurin signal transduction pathways. Mol Microbiol.

[44] Wang H, Liang Y, Zhang B, Zheng W, Xing L, Li M (2011). Alkaline stress triggers an immediate calcium fluctuation in *Candida albicans* mediated by Rim101p and Crz1p transcription factors. FEMS Yeast Res.

[45] Petrezsélyová S, López-Malo M, Canadell D, Roque A, Serra-Cardona A, Marqués MC, et al. Regulation of the Na+/K+−ATPase Ena1 expression by calcineurin/Crz1 under high pH stress: a quantitative study. PLoS One. 2016; doi:10.1371/journal.pone.0158424. 10.1371/journal.pone.0158424 PMC4928930 27362362

[46] Thewes S (2014). Calcineurin-Crz1 signaling in lower eukaryotes. Eukaryot Cell.

[47] Wang Z, Wilson WA, Fujino MA, Roach PJ (2001). Antagonistic controls of autophagy and glycogen accumulation by Snf1p, the yeast homolog of AMP-activated protein kinase, and the cyclin-dependent kinase Pho85p. Mol Cell Biol.

[48] Ruiz A, Serrano R, Ariño J (2008). Direct regulation of genes involved in glucose utilization by the calcium/calcineurin pathway. J Biol Chem.

