# Supplementary material for: Regulation of the reserve carbohydrate metabolism by alkaline pH and calcium in Neurospora crassa reveals a possible cross-regulation of both signaling pathways
Source: BMC Genomics. 2017 Jun 9;18:457. doi: 10.1186/s12864-017-3832-1 (PMC5466789; doi:10.1186/s12864-017-3832-1)
Supplement: Supplementary file 1 — Oligonucleotides used in this study. (DOCX 29 kb) [file 12864_2017_3832_MOESM1_ESM.docx]

**Table S1.** *Oligonucleotides used in this study*.

| **Primer** | **Sequence (5’→3’)** | **Source** | **Name** | **Position ^a^** |
| --- | --- | --- | --- | --- |
| *qPCR* | | | | |
| qPac3-F | CAAGCATCGACCCGTATCAT | NCU00090 | − | +1203 to +1222 |
| qPac3-R | TGGTGAGTGACCCGAAGTA | NCU00090 | − | +1312 to +1330 |
| qGSN-F | TACCAAGCATCACCACCAACCTCT | NCU06687 | − | +1541 to +1564 |
| qGSN-R | TGTCTGCGGCTCTTCTGGGTAAAT | NCU06687 | − | +1689 to +1712 |
| qRAMIF-F | TCTGCGATGCCGAGTTGT | NCU05429 | − | +1487 to +1504 |
| qRAMIF-R | ACTCGTTGCCCTCGAAGT | NCU05429 | − | +1616 to +1633 |
| qGNN-F | ACAAGCACCCGAACCCAC | NCU06698 | − | +1134 to +1151 |
| qGNN-R | AAGGGTGGGCGATGCTGT | NCU06698 | − | +1234 to +1251 |
| qGPN-F | TGCCAATATCGAAATCACCCGCGA | NCU07027 | − | +2247 to +2270 |
| qGPN-R | TCTCGATGGCCTCAAACACCTTGA | NCU07027 | − | +2375 to +2398 |
| qDESRAM-F | TCGGCGGTAATCAAGCCA | NCU00743 | − | +3794 to +3811 |
| qDESRAM-R | TGAATTTGCCGGCTTCGT | NCU00743 | − | +3950 to +3967 |
| qtps1-F | GGTTGACTTCCTCATGGTGGTTG | NCU00793 | − | +2529 to +2551 |
| qtps1-R | GTCGCCTTAGCCTCAGTGTTTCT | NCU00793 | − | +2647 to +2669 |
| qtps1alfa-F | TGCCGAGCTGACAAGGATAAGC | NCU09715 | − | +1410 to +1431 |
| qtps1alfa-R | GGCATCCGTCACACCCTCAATA | NCU09715 | − | +1512 to +1533 |
| qtps2-F | CGAGATCAAGCCCGAGAACTGC | NCU05041 | − | +2817 to +2838 |
| qtps2-R | CATCCTCAGGTTCCAACAAGTGCC | NCU05041 | − | +2885 to +2908 |
| qtre1-F | CAAGAGGGCCGACATCACTATGG | NCU00943 | − | +1944 to +1966 |
| qtre1-R | CCCCTTCCTGGCGAACTTCTT | NCU00943 | − | +2011 to +2031 |
| qtre2-F | CGCTCGGCACTCTGACTCC | NCU04221 | − | +2153 to +2171 |
| qtre2-R | CTCAGAGAGCGCCTTGTTCCG | NCU04221 | − | +2206 to +2226 |
| qcrz1-F | AATGGACCCCTTTGTGCTCCC | NCU07952 | − | +1881 to +1901 |
| qcrz1-R | GACATCATCGTAGTCGCTAGCTTCG | NCU07952 | − | +2004 to +2028 |
| 4054Tub-F | CCTCCACCTTCGTCGGTAACTCC | NCU04054 | − | +1091 to +1113 |
| 4054Tub-R | GGTACTGCTGGTACTCGGAGACG | NCU04054 | − | +1254 to +1276 |
| 4173ACT-F | CCATGTACCCTGGTCTCTCCGAC | NCU04173 | − | +911 to +933 |
| 4173ACT-R | CCACCGATCCAGACGGAGTACTTG | NCU04173 | − | +1005 to +1028 |
| *ChIP-PCR* | | | | |
| PacC-F | GACCCAACAGCCCAACTT | p*gsn* | *gsn* | -1918 to -1901 |
| SREBP-RP2 | TCTGACCTTTCCCAATCAG | p*gsn* | *gsn* | -1645 to -1627 |
| pGPNNit-F2 | CTGGCTGGCTCCGTCTTA | p*gpn* | *gpn* | -725 to -708 |
| pGPNNit-R2 | GAGGTAAGTGGGGCAGTC | p*gpn* | *gpn* | -527 to -510 |
| gnnPAC3-Fp | CTTGGGGCTCTCTCGTCTGTG | p*gnn* | *gnn* | -380 to -360 |
| gnnPAC3-Rp | CAGTCGAAGGAGGCTGCAGTG | p*gnn* | *gnn* | -288 to -268 |
| branch-FP5 | TAAATGGGAAGCTAGAAGGGACGAC | p*gbn* | *gbn* | -973 to -949 |
| branch-RP5 | GGAGCTTCAACAAACACGGACATG | p*gbn* | *gbn* | -747 to -724 |
| DEBp-F2 | GCCTGTTTTCTGACGGGT | p*gdn* | *gdn* | -692 to -675 |
| DEBp-R2 | TTGGCTGTGATAGGACCG | p*gdn* | *gdn* | -536 to -519 |
| tresynt-Fp | GCTCAAGTTCCTCAGCGCTACATT | p*tps-1* | *tps-1* | -856 to -833 |
| tresynt-Rp | CTTGACATCTTCTGCCCAGACACA | p*tps-1* | *tps-1* | -727 to -704 |
| alfatre-Fp | CGACAACCCGCCAATCAGC | p*tps-1*α | *tps-1α* | -547 to -529 |
| alfatre-Rp | AAGATCGGAAACTTGGAATGGCTGG | p*tps-1*α | *tps-1α* | -351 to -327 |
| trephosp-Fp | GATCTGTTTACATCCTTCGTCCCGC | p*tps-2* | *tps-2* | -119 to -95 |
| trephosp-Rp | TGTTGTCCTTGTGTCATCTTGGCG | p*tps-2* | *tps-2* | -7 to +17 |
| tre1-Fp | CACACATTTCTCAGTCTCGTTCCCC | p*tre-1* | *tre-1* | -737 to -713 |
| tre1-Rp | GCTTGTTCCCCTGCTTCCCT | p*tre-1* | *tre-1* | -586 to -567 |
| tre2-Fp | CCAGCCTCATCTGCGTCCT | p*tre-2* | *tre-2* | -1064 to -1046 |
| tre2-Rp | TGGCGATGGAAAGCGGGAT | p*tre-2* | *tre-2* | -942 to -924 |
| qUbi-F | CGAGTCTTCGGATACGATTG | NCU05995 | *ubiquitin* | +805 to +824 |
| qUbi-R | CCATCCTCCAACTGCTTAC | NCU05995 | *ubiquitin* | +894 to +912 |

^a^Primers are positioned according to the ATG start codon from cDNA (qPCR) or genomic DNA (ChIP-PCR).
