# Supplementary figures and images for: Regulation of the reserve carbohydrate metabolism by alkaline pH and calcium in Neurospora crassa reveals a possible cross-regulation of both signaling pathways
Source: BMC Genomics. 2017 Jun 9;18:457. doi: 10.1186/s12864-017-3832-1 (PMC5466789; doi:10.1186/s12864-017-3832-1)

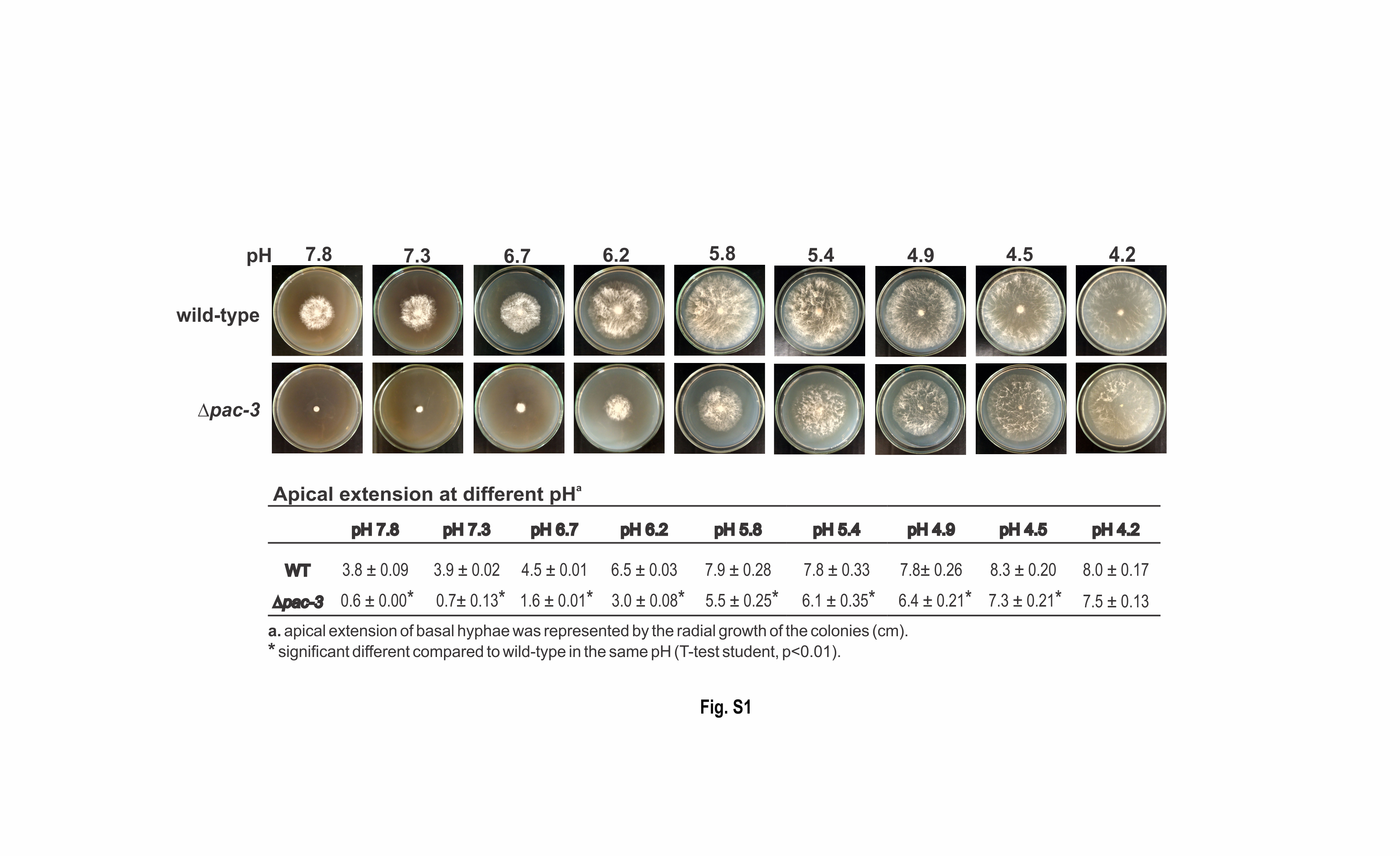

Supplement: Supplementary file 2 — Morphological analyses of the wild-type and Δpac-3 mutant strains under different pH conditions. The strains (107 conidia) were inoculated onto Petri dishes containing solid VM medium plus 2% sucrose from pH 4.2 (acid condition) to pH 7.8 (alkaline condition) at 30 °C. Images of colony morphology were captured after 24 h. Apical extension was measured in centimeters and is shown in the table below. The results represent at least two independent experiments in duplicate. The asterisks indicate the significant difference between wild-type and mutant strains at the same pH (Student’s t-test, P < 0.01). (TIFF 3061 kb) [file 12864_2017_3832_MOESM2_ESM.tif]

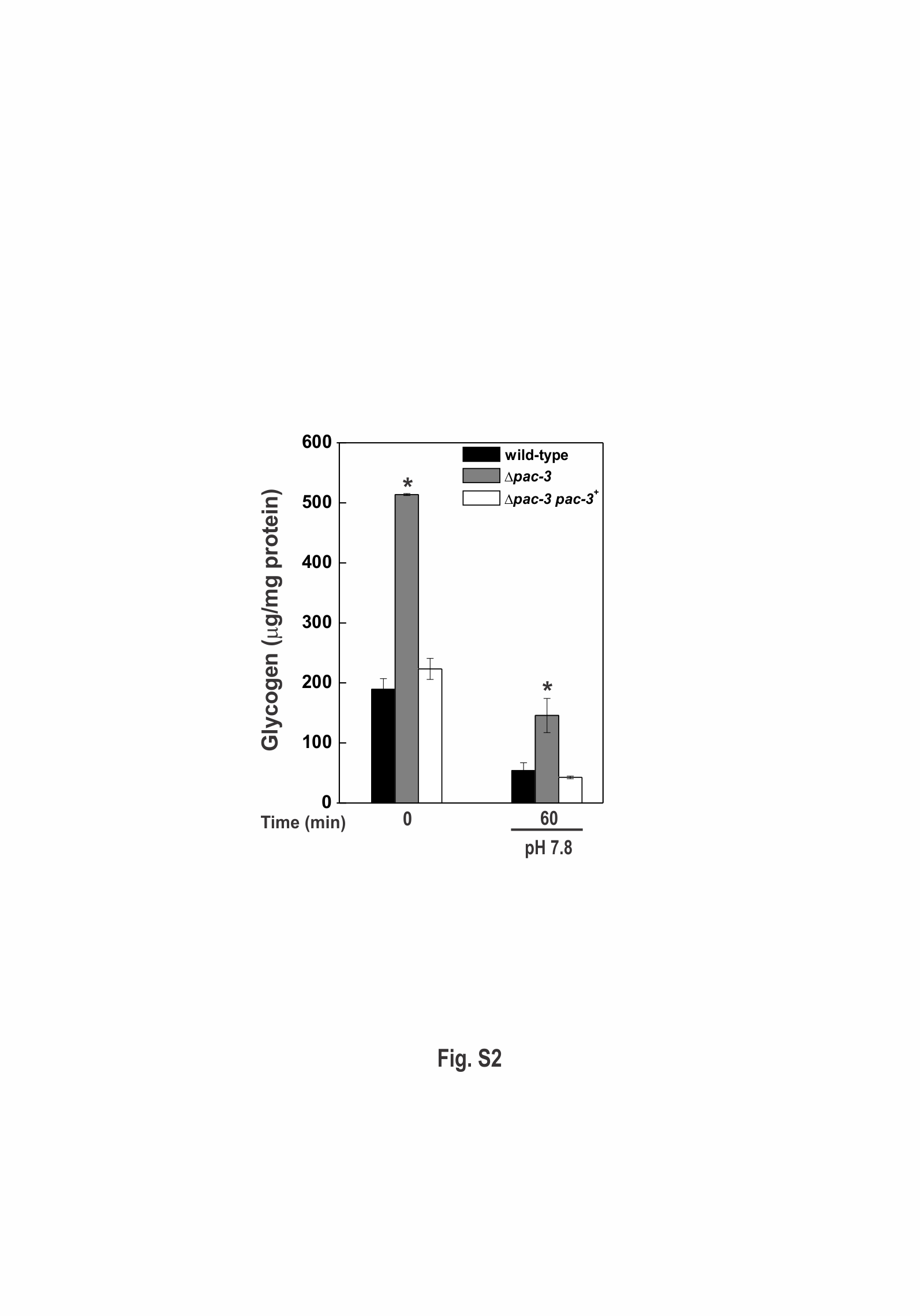

Supplement: Supplementary file 3 — Glycogen quantification in the wild-type, Δpac-3 mutant and Δpac-3 pac-3 + complemented strains at normal growth pH (5.8) and alkaline pH (7.8). Mycelial samples cultured at pH 5.8 (zero) at 30 °C for 24 h and shifted to pH 7.8 for 1 h were used for glycogen quantification. The asterisks for the Δpac-3 data indicate significant differences compared to the wild-type strain at the same condition (Student’s t-test, P < 0.01). The results represent the average of three independent experiments. Bars indicate the standard deviation from the biological experiments. (TIFF 317 kb) [file 12864_2017_3832_MOESM3_ESM.tif]
