# Supplementary material for: Regulation of the reserve carbohydrate metabolism by alkaline pH and calcium in Neurospora crassa reveals a possible cross-regulation of both signaling pathways
Source: BMC Genomics. 2017 Jun 9;18:457. doi: 10.1186/s12864-017-3832-1 (PMC5466789; doi:10.1186/s12864-017-3832-1)
Supplement: Supplementary file 4 — Multiple sequence alignment of the N. crassa protein codified by the ORF NCU07952 and the CrzA proteins from A. fumigatus (XP_750439.1) and A. nidulans (BAE94327.1), and the CRZ-1 protein from Trichoderma reesei (ETS01683.1). The C2H2 zinc finger DNA binding domain at the C-terminus is highlighted by asterisks and the putative calcineurin interaction site is indicated by an upper line. ClustalW (http://www.ebi.ac.uk/Tools/msa/clustalw2/) was used for sequence alignment. Identical amino acids are shaded in black and conserved amino acids are shown in gray. (DOC 48 kb) [file 12864_2017_3832_MOESM4_ESM.doc]

*A.fumigatus* 1 -MASQEMFPELGQSPAPGVKSRG--VSRSPHPHQQQQQQQHQQHQGQFTGTVTGLDLDSSIATASSFANSSFDPNSNNVS
*A.nidulans* 1 -MDPQDTLQDLGQAPAAHIN-RS--ASPSAHAHQQYNNNHN------------DLTIDPSVTSNSSYPPSSFANNS---A
*T.reesei* 1 ----MAHEPQRGRSPSAG---------GFQSDINQSHSPARSPLAPTNEQPSAGLGVGLGVDLDSSQQQQQLQQQQQQQQ
*N.crassa* 1 --MDQQYTDARGRSLSAASTGGGQVHDQQQQSHIRNHSPSPTPFPNSNDGVNNGLGLGLIDPSASQHFQPEFSYGGPNPF


*A.fumigatus* 78 PSAESYGYTAAGYLSGTPASQTDQNYAN-SLQIPQSYGTGLVPQFNESRGLPIQQQSQQQHHQQPSLDDNFSDLLNSNAT
*A.nidulans* 62 PGSEAFAYSSS---YLTPATATDHNFARPSLQIPQSFDQGLSHQP---------------------AEENFSNLLNSNTG
*T.reesei* 68 QQRLQQQQQRAFAAPLHPNYDSFGANGFLGAQANAVDPTNGFDPSASFG----QQPATGPDSTLSLNAQAQHNYLSPNLH
*N.crassa* 79 QQHSPFSQPGLDFNQGYTNQLENQDNSFGGLSQPAYSPN----------------------------------LMASNFG


*A.fumigatus* 157 EYDFNTVYQTHSPSSNTAPEYDSSLLLDPQVHQQSHP------TQIPSSHSSTSPQISPLEQQQHSSPGPMSTQGSTTVA
*A.nidulans* 118 DFDFSLYQGSSP--NNTGSDYPSSGLLDPQQSGNQAVNPVDLVSQIPSPHPSNSSQTSPLDQPPSS---AMSPPASSPGT
*T.reesei* 144 DGDFSLFPSAAE----QGDQ-YNAPLFEQPPLGD------------LNAMTSPHSHQSPTPPQLFQPDSLQSPPFNRHQF
*N.crassa* 125 DADYGIFPTTTA----AGQFNGSLFITDNQSINN------------PDPNMMAQGSHSPEPPHLLSPEINSPAFAQGRFP


*A.fumigatus* 231 YYTPQHSRHASLDPATAAFLTSNTHPDWQAVMGNSAAFQGHRRAPSEVSEISSAAPSPYLSQHESFDGV--DNNPSPLLA
*A.nidulans* 193 FYTPQHSRHTSLDPASAAYMTNVSHPEWQAVMNN-SAFHGHRRAPSEVSEVSSAAHSPYLPQHDSFDVA--DNNPSPLLA
*T.reesei* 207 SSPPTHSRNASLGPEAALLPS-------QIGDWTQPQFQGHRRTPSEYSDVSSVAPSPHLVSSDTFDAD--QSGHSPLQR
*N.crassa* 189 MATGRHSRNASLGPEAALLPG-------QD-WSHMPQFQGHRRSASELSDVSSVAHSPNLGGLDSFDPI--ENNHSPLQG


*A.fumigatus* 309 PQNDPSLYD-SALGIENFTLSEQHQQHQG-FSPAHSPYISPRLMPQQGQEMMPNVP--YLSGPAPNTQYPTPPNDMYGNG
*A.nidulans* 270 AQNDPSLYDNAALGIESFTLSEHHQPQTQGISPHHSPYISPQLMPQHPTDIIPGGP--FISAPATNSAYPTPPTEGYPNG
*T.reesei* 278 PA-DVSLYQ-EVLGIGSFSLADHGSPGYHGRSPSHSPAISPRIMPQQMPDTMQPS----FNLIPPNGGFDGVSGYPDLQP
*N.crassa* 259 PQ-ADALYS-QLNGISNFSLSDD----HIGRSPSHSPAVSPRIHPQQSPDEIDPNQPNHFMLHTPANSFG--PPATYMQP


*A.fumigatus* 385 AEGMMNMSQGTHPSVDIGQASQMAPPSINVEFAPP-SRIPSFGPSKPASNLDSLSPPPSSTRSRGRSKSDPYAHPSTSRL
*A.nidulans* 348 GD--------------IGQASQMAPPSINVEFAPP-AKAQVFPPEKSTADMDSLSPPPSLRTSRMRSKSDPYAVSISRPR
*T.reesei* 352 SHESFPSLSG----GMGGDMHQMAPPAINIDFAPTNSRQGSFEPPKSQMDQDSLTPPERRPKSRPRAVTDPFHPGSGILP
*N.crassa* 331 QQEAFPQLSLDDPSGMQAQQNMPAPPAINIDFAPAPAKSG-LDQPTNLDNNSLALPNRARGRMRPRAVTDPFN-NSGYRS


*A.fumigatus* 464 RSSSTSSSLDPLAPTTPRSLSPFDSFGRQQQSNPSSRDPSPSRSNRRLSTSSIDSRNYILGLADPQRPGASPND------
*A.nidulans* 413 SPSSPSASLDALAASSPRSLSPFN-VGRHPYSNPSSREPSPARSARRLSTSSVDSRNYILGLADPQRPGSNNTD------
*T.reesei* 428 PGNLGSSLGVDLAARSDTASRSLSPLDRSGTSSPASR--------RRQSTSSVPNNVIALRLADPEYQNSQEAG------
*N.crassa* 409 PSPSGSLSPSSAADLRPSSARSLSPMDRSGAGSINSR--------RRQSTSSVPNNVIALRLADPNYNGSGENGG-----

 *******************************************************
*A.fumigatus* 538 --SKRVQKHPATFQCNLCPKRFTRAYNLRSHLRTHTDERPFVCTVCGKAFARQHDRKRHEGLHSGEKKFVCQGELSRG-G
*A.nidulans* 486 --SKRVQKHPATFQCTLCPKRFTRAYNLRSHLRTHTDERPFVCTVCGKAFARQHDRKRHEGLHSGEKKFVCRGDLSRG-G
*T.reesei* 494 -TSKRMQKHPATFQCTLCPKRFTRAYNLRSHLRTHTDERPFVCTVCGKAFARQHDRKRHESLHSGEKKFVCKGDLKTG-G
*N.crassa* 476 -GPRRAQKHPATFQCKVCPKRFTRAYNLRSHLRTHTDERPFKCTVCDKAFARQHDRKRHEGLHSGEKKFICKGELPVAGQ


*A.fumigatus* 615 QWGCGRRFARADALGRHFRSEAGRICIKPLLDEESQERERSLMDQQQHHLQPLPQQ-----VMVP---VDNPHAGNFVLP
*A.nidulans* 563 QWGCGRRFARADALGRHFRSEAGRICIKPLLDEESQERERTLINQQQQHLQPVNQP-----LMLPGQGTEAQHTGSFILP
*T.reesei* 572 QWGCGRRFARADALGRHFRSEAGRICIKPLLDEEMVERQRQWQEQRMQQNMAQNMANPQVMGMDAGPAYPMDASGNYTLP
*N.crassa* 555 QWGCGRRFARADALGRHFRSEAGRICIRPLLEAENRERQRQYAEAMQNAAQGMMQQQGGMMMSPGMDPNGEFQMDPFVLP


*A.fumigatus* 687 AALLAQYPALQTLQWDQIAASADDPSDIGGRSSFDASSGNEFGFEDDDSGLSSVSGINAGYSAAGNFY------------
*A.nidulans* 638 AALLAQYPALQTLQWDQIPAGTDDTSDIGGRNSFDASSGGEFGFDDDESGISVSGMSTGYASDQGNIYNVDAQGQMLGVN
*T.reesei* 652 QALLAQYPALAQMNWSATDMGGGLDDELSGRSSFDASDYDDGDDGGYISSSGARYPEEGMSQNYADMNYMGDYGR-----
*N.crassa* 635 QALLAQYPALALLPAGPAAMGDGAGLEEDLGSNYEASDYDDVEEGGYVSGPGTGFGPGSMQEGYGELGYASDYGGR----


*A.fumigatus* -------------
*A.nidulans* 718 PGEAGYANPNWGK
*T.reesei* -------------
*N.crassa* -------------

**Fig. S3**
